# Skin microbiome variation in people living with HIV: associations with antiretroviral therapy and host factors

**DOI:** 10.3389/fcimb.2026.1794878

**Published:** 2026-04-15

**Authors:** Muhammad Anshory, Nikolaos Strepis, Milanitalia Gadys Rosandy, Aulia Rahmi Pawestri, Agustin Iskandar, Indah Adhita Wulanda, Lita Setyowatie, Nathanael Ibot David, Natalia Rasta Malem, Handono Kalim, Tamar E. C. Nijsten, Jan L. Nouwen, Hok Bing Thio

**Affiliations:** 1 Department of Dermatology, Erasmus University Medical Centre (Erasmus MC), Rotterdam, Netherlands; 2 Department of Internal Medicine, Faculty of Medicine, Universitas Brawijaya, Malang, Indonesia; 3 Department of Pathology, Erasmus University Medical Centre (Erasmus MC), Rotterdam, Netherlands; 4 Department of Parasitology, Faculty of Medicine, Universitas Brawijaya, Malang, Indonesia; 5 Department of Clinical Pathology, Faculty of Medicine, Universitas Brawijaya, Malang, Indonesia; 6 Department of Dermatology and Venereology, Faculty of Medicine, Universitas Brawijaya, Malang, Indonesia; 7 Department of Medical Microbiology and Infectious Diseases, Erasmus University Medical Centre (Erasmus MC), Rotterdam, Netherlands

**Keywords:** 16S rRNA sequencing, antiretroviral therapy (ART), HIV infection, microbial diversity, skin microbiome

## Abstract

**Introduction:**

The skin microbiome plays a key role in cutaneous immunity and is shaped by host immune status. HIV infection is associated with immune dysfunction and dermatological disease, yet its impact on the skin microbiome and the modifying effect of antiretroviral therapy (ART) remain incompletely defined. This prospective observational study conducted in Indonesia aimed to characterize differences in skin microbiome composition across HIV status and ART exposure and relate these profiles to clinical parameters.

**Methods:**

Skin swabs were obtained from sebaceous (posterior neck) and dry (dorsal forearm) sites in HIV-ART-naïve individuals, people living with HIV on ART, and HIV-negative controls, and analyzed using 16S rRNA gene sequencing. Microbial diversity and community structure were assessed using Bray–Curtis dissimilarity, PERMANOVA, and differential abundance testing with ANCOM-BC2, with multivariable models adjusting for demographic, clinical, behavioral, and anatomical factors and subgroup analyses by body mass index, skincare habits, and sampling site.

**Results:**

In total, 488 samples from 244 participants were analyzed. Both HIV groups showed significantly reduced alpha diversity compared with controls, and overall community composition differed by HIV status, although sampling site explained a larger proportion of variation. Across groups, the microbiome was dominated by *Corynebacterium, Cutibacterium, Staphylococcus*, and *Streptococcus*. Differential abundance analyses indicated targeted genus-level shifts rather than global dysbiosis, with ART-naïve individuals showing the most consistent deviations, including increased *Staphylococcus* and reduced *Streptococcus* relative to controls, and partial attenuation among participants receiving ART. HIV-associated differences were observed within both sebaceous and dry sites, and HIV status remained independently associated with microbiome composition after adjustment.

**Conclusions:**

These findings suggest that HIV infection is associated with subtle but consistent alterations in the skin microbiome within the context of strong site-specific skin microenvironments. Longitudinal studies integrating functional profiling and host markers of cutaneous barrier integrity and inflammation are needed to clarify their clinical implications.

## 1 Introduction

The skin, the outermost part of human body, serves as a crucial innate immune barrier in humans. The primary function of the skin is to form a physical, chemical, and immunological protective barrier between the body and the external environment (Abdallah et al., 2017). The skin is composed of stratified keratinized epithelium, which undergoes terminal differentiation to acquire a strong structure. The normal skin surface is an acidic, high-salt, dry, and aerobic environment, but the follicle-sebaceous units are relatively anaerobic and much richer in lipids. The skin microbiome is an integral component of this barrier, contributing to immune maturation, maintenance of epidermal integrity, and colonization resistance against pathogens. Commensal microbes can modulate local innate and adaptive immune responses (for example via antimicrobial peptide induction and competition for ecological niches), while host factors such as sebum composition, pH, hydration, and inflammatory tone shape microbial community structure across different skin microenvironments (Grice and Segre, 2011; Smythe and Wilkinson, 2023; Xia et al., 2025). Disruptions in the skin microbiome (dysbiosis) are linked to various skin disorders such as acne, psoriasis, and dermatitis. Factors like systemic antimicrobial treatments can alter the skin microbiota, making it essential to understand what influences its balance to maintain skin health (Lee and Kim, 2022; Borrego-Ruiz and Borrego, 2024).

Human Immunodeficiency Virus (HIV) significantly impacts skin structure, immune responses, and the microbiome, contributing to diverse dermatological conditions. HIV infection can also affect the human microbiome. As HIV progresses to AIDS, patients often develop skin-related conditions like prurigo and Kaposi’s sarcoma, suggesting a link between immune deficiency, skin disorders, and microbiome imbalance (Ogai et al., 2023; Anshory et al., 2025).

Mechanistically, HIV-associated immune dysfunction may alter skin immunity and barrier homeostasis, potentially reshaping the skin microbial ecosystem. Reduced immune surveillance, chronic inflammation, and increased susceptibility to bacterial and fungal infections may shift the balance between commensal and opportunistic taxa (Chimbetete et al., 2023; Anshory et al., 2025). Antiretroviral therapy (ART) partially restores immune function and reduces systemic inflammation (Hileman and Funderburg, 2017), but whether and to what extent ART normalizes HIV-associated skin microbiome alterations remains unclear. Because skin microbiota are strongly structured by local microenvironmental conditions, evaluating distinct microenvironments (for example sebaceous versus dry sites) is critical to disentangle HIV/ART-associated signatures from site-driven variation (Schommer and Gallo, 2013; Suri et al., 2025). However, research exploring the interaction between HIV-induced immune suppression, skin conditions, and microbiome composition is still limited.

The current study aimed to characterize differences in the skin microbiome among untreated HIV-infected individuals, individuals receiving antiretroviral therapy (ART), and HIV-negative healthy controls. Furthermore, by correlating microbiome profiles with a range of relevant clinical parameters, including CD4+ T cell counts and the presence of skin disorders, we aimed to advance our understanding of the potential clinical significance and underlying mechanisms of skin microbiome dysbiosis in the HIV population. The results may be used to guide future diagnostic, prognostic, and therapeutic approaches that are designed to improve dermatological health and the overall quality of life for people living with HIV.

## 2 Materials and methods

### 2.1 Study approach and cohort selection

This investigation was structured as a prospective, observational study aimed at comparing the clinical features and microbiome composition of three separate participant cohorts. Participants were enrolled from both the inpatient and outpatient departments of Dr. Saiful Anwar Regional General Hospital in Malang, Indonesia. A consecutive sampling strategy was employed to recruit individuals, who were then stratified into one of three groups:

- HIV-ART-Naïve Group: This cohort comprised individuals with a new HIV diagnosis (WHO stages 1-4) who were either untreated with antiretroviral (ARV) agents or had received such therapy for under two weeks. The group also included patients who had ceased ARV treatment for a period exceeding six months.
- HIV-on-ART Group: This group was composed of HIV-positive patients with a documented history of continuous ARV therapy for at least one year.
- HIV-Negative Control Group: A control group was formed of individuals confirmed to be HIV-negative upon enrollment to provide a comparative baseline.

### 2.2 Ethical framework and study timeline

All procedures adhered to stringent ethical guidelines. Prior to commencement, the study protocol was reviewed and granted full approval by the Institutional Ethics Committee of Dr. Saiful Anwar General Hospital, Malang (reference number 400/135/K.3/102.7/2023, 5 Juni 2023). Participant enrollment occurred between October 2023 and June 2024. Informed written consent was obtained from all participants following a detailed explanation of the study’s objectives and procedures. To protect participant confidentiality, all questionnaire data were anonymized and managed within a secure, limited-access electronic database. The international transfer of biological specimens to Erasmus MC was governed by a Material Transfer Agreement (MTA), which was officially sanctioned and monitored by the Health Development Policy Agency of the Indonesian Ministry of Health (BKPK Kemenkes RI) reference number FK.06.01/H/5614/2024.

### 2.3 Procedures for data and specimen collection

To maintain data quality and consistency, all data and sample collection were carried out by trained research staff following standardized protocols.

#### 2.3.1 Collection of clinical and demographic information

Demographic details and a comprehensive medical history were gathered from each subject via a validated, structured electronic questionnaire. The variables gathered included: demographic factors (age, sex, place of residence, occupation, marital status, education, income), HIV-related history (date of diagnosis, suspected transmission route), and lifestyle habits (skin care habit practices).

Skin care habits were assessed using a previously validated skin care habit questionnaire (Hahnel et al., 2019), which collected information on the use of skin care products, frequency of face washing, bathing, and shampooing, as well as the frequency and anatomical location (face and neck, body) of leave-on product application. The full list of questionnaire parameters is provided in **Supplementary Table 1**. Any presenting symptoms were also documented.

Specialist physicians from the Department of Dermatology and Venereology conducted a thorough physical, and dermatological examination on each participant. Any identified cutaneous manifestations were diagnosed, with their type and anatomical location meticulously recorded. The primary diagnosis, co-existing medical conditions, and the clinical setting (inpatient vs. outpatient) were also noted. A detailed inventory of all current and previous medications, encompassing treatments for HIV, dermatological issues, and other health conditions, was compiled for each individual.

#### 2.3.2 Blood collection and laboratory testing

Venous blood was collected from each participant by a certified phlebotomist. These samples were then transferred to the hospital’s main laboratory for quantification of CD4+ T-cell counts.

#### 2.3.3 Collection of skin microbiome samples

Skin microbiome samples were collected from two distinct anatomical sites representing different skin microenvironments, as previously described in other studies (Grice et al., 2009; Ogai et al., 2018). Two locations were selected to represent sebaceous and dry skin characteristics. Skin swab samples were obtained from the sebaceous posterior neck and the dry dorsal forearm. For each site, a 4 × 4 cm area was demarcated. A sterile COPAN FLOQSwab^®^ (Copan Italia s.p.a., Italy), pre-moistened with a saline solution, was used for sampling. The swab was rubbed firmly across the designated skin area for 30 seconds using a Z-shaped motion. It was then turned over, and sampling continued for an additional 30 seconds with strokes perpendicular to the initial pattern to maximize microbial recovery. The swab tip was then aseptically broken off into a tube containing COPAN eNAT^®^ (Copan Italia s.p.a., Italy) transport and preservation medium and stored at −20°C.

### 2.4 Microbiome analysis

#### 2.4.1 DNA extraction

Microbial DNA was extracted from all skin samples at the Laboratory of Parasitology, Faculty of Medicine, Universitas Brawijaya, using the QIAamp DNA Microbiome Kit (QIAGEN, Netherlands), following the manufacturer’s protocol optimized to enrich microbial DNA and reduce host genomic DNA contamination. The protocol included benzonase treatment to degrade host DNA, mechanical cell lysis by bead beating, protein digestion with proteinase K, and purification using silica spin columns. Columns were washed with AW1 and AW2 buffers, and DNA was eluted in 50 μL of buffer AUE. All DNA extracts were stored at −20 °C until further processing.

#### 2.4.2 Sample transport and next-generation sequencing

Extracted DNA samples were transported to the Erasmus University Medical Center (Erasmus MC), Rotterdam, the Netherlands, using a professional international courier service with temperature-controlled cryobox packaging to maintain a constant temperature of −20 °C during shipment. At the Department of Clinical Genetics, Erasmus MC, next-generation sequencing was performed, including amplification of the hypervariable regions of the 16S rRNA gene using universal primers, ligation of molecular barcodes for multiplexing, purification of amplicons with magnetic beads, and sequencing on an Illumina high-throughput platform (Illumina, San Diego, CA, USA).

#### 2.4.3 Bioinformatic processing and data preparation

Initial processing of raw sequencing reads, including quality filtering and taxonomic assignment, was conducted at Erasmus MC. Amplicon sequence variant (ASV) count tables and taxonomy were imported into R (v4.5.2) and integrated with sample-level demographic and clinical metadata. Taxonomic classification was performed using the SILVA 16S rRNA database (release 138.1) (Quast et al., 2012).

To ensure high data quality, a multi-step filtering and decontamination pipeline was applied. Potential contaminant ASVs were identified and removed using the decontam package, based on their prevalence in negative controls and their inverse correlation with DNA concentration. The dataset was further refined by excluding: ASVs present in fewer than 1% of samples, ASVs lacking taxonomic annotation, and samples with zero remaining reads. This resulted in a curated dataset suitable for downstream ecological and differential abundance analyses.

#### 2.4.4 Statistical analysis

##### 2.4.4.1 Clinical and baseline characteristics

Descriptive statistics were used to summarize cohort characteristics. Differences in categorical variables were assessed using the Chi-square test, while continuous variables were compared using independent t-tests or ANOVA for normally distributed data, and Mann-Whitney U or Kruskal-Wallis tests for non-normally distributed data.

##### 2.4.4.2 Microbiome community analysis

Alpha diversity was assessed at the ASV level using the Observed richness index and compared across clinical groups using the Kruskal-Wallis test. Beta diversity was assessed on relative abundance-transformed ASV data using Bray-Curtis dissimilarity and visualized by principal coordinates analysis (PCoA). Group-level differences in community composition were tested using permutational multivariable analysis of variance (PERMANOVA) with 999 permutations, with both p-values and explained variance (R²) reported.

Additional univariable PERMANOVA models were performed to evaluate associations between microbial community composition and individual demographic and clinical variables. Multivariable PERMANOVA models incorporating key host factors were fitted to better capture clinically relevant correlations, while social and economic variables were not included in the multivariable models. PCoA panels were generated to visualize multivariable effects.

##### 2.4.4.3 Identification of microbial signatures and relative abundance profiles

Differential abundance was assessed using ANCOM-BC2, with HIV status specified as the fixed effect. Taxa with low prevalence and samples with low sequencing depth were excluded prior to analysis. Multiple testing correction was applied using the Holm method, with statistical significance defined at α=0.05. Structural zeros were accounted for, negative lower bounds were allowed, and a global test across groups was performed. Differentially abundant taxa were annotated taxonomically and summarized at the genus level for visualization.

For visualization, ASVs identified as differentially abundant in at least one group contrast were aggregated to the genus level. Genus-level log fold changes (LFC) were calculated as the mean ASV-level LFC within each genus, and differentially abundant genera were visualized using waterfall plots.

Genus-level relative abundance analysis was performed following taxonomic aggregation and transformation to relative abundances. The 20 most abundant genera across all samples were identified, with remaining genera collapsed into an “Other” category. Relative abundance profiles were visualized using stacked bar plots stratified by clinical group and are intended for descriptive visualization, whereas formal statistical inference relied on PERMANOVA and differential abundance analysis using ANCOM-BC2.

##### 2.4.4.4 Subgroup and multivariable analyses

Subgroup analyses were conducted using a standardized R pipeline across strata defined by body mass index (BMI), skincare habit score, and sampling site. Within each subgroup, differential abundance, and genus-level relative abundance were assessed using identical analytical workflows. BMI and skincare habit score were dichotomized using cohort medians (22.8 kg/m² and score 12, respectively). Sampling site analyses compared neck and elbow samples. Subgroup analyses included both direct between-stratum comparisons and analyses restricted to participants receiving ART to better capture subgroup-specific variation.

All analyses were conducted in RStudio (R v4.5.2) using phyloseq (v1.52.0), vegan (v2.7.1), ANCOMBC (v2.10.1), ggplot2 (v4.0.1), and decontam (v1.28.0), with data manipulation performed using dplyr (v1.1.4) and tidyr (v1.3.1).

## 3 Results

### 3.1 Baseline characteristics

A total of 490 skin microbiome samples were obtained from 245 individuals. One HIV-negative control participant was excluded due to a sampling error, resulting in a final cohort for analysis of 488 skin microbiome samples from 244 individuals, including 130 people living with HIV (115 HIV-on-ART and 15 HIV-ART-naïve) and 114 HIV-negative healthy controls.

Participants in the HIV-ART-naïve arm were younger (mean ± SD: 31.8 ± 7.79 years) than those in the HIV-on-ART (42.31 ± 10.5) and healthy-control (40.15 ± 11.09) groups (p=0.001). The proportion of women did not differ significantly across groups (46.7%, 41.7%, and 54.4%, respectively; p=0.159). BMI differed significantly between groups, with median values of 23.19, 21.47, and 24.18 in the HIV-ART-naïve, HIV-on-ART, and control groups, respectively (p=0.001). Marital status distributions also differed significantly (p=0.013), with higher rates of divorce and widowhood observed in the HIV-on-ART group. Educational attainment and monthly income likewise varied across study arms (both p<0.05).

Hematologic and immunologic markers increased stepwise from HIV-ART-naïve through HIV-on-ART to healthy controls: hemoglobin and CD4+ T cell counts were both highly significant (p<0.001). Skin disease prevalence was highest in the HIV-ART-naïve group (66.7%) and lowest among healthy controls (8.8%; p<0.001). The complete baseline characteristics are presented in **Table 1**.

**Table 1:** Baseline characteristics.

Variable	Control	HIV-ART-naïve	HIV-on-ART	P
n	114	15	115	
Age, mean (SD)	40.15 (11.09)	31.80 (7.79)	42.31 (10.50)	0.001
Sex=Woman, n (%)	62 (54.4)	7 (46.7)	48 (41.7)	0.159
BMI, median (IQR)	24.18 [21.25, 26.21]	23.19[22.01, 24.55]	21.47[19.26, 24.14]	<0.001
Marriage status, n (%)				0.013
Single	23 (20.2)	6 (40.0)	31 (27.0)	
Married	83 (72.8)	9 (60.0)	61 (53.0)	
Divorce	3 (2.6)	0 (0.0)	12 (10.4)	
Widowed	5 (4.4)	0 (0.0)	11 (9.6)	
Education, n (%)				<0.001
Elementary School	7 (6.1)	4 (26.7)	25 (21.7)	
Junior High School	12 (10.5)	1 (6.7)	12 (10.4)	
Senior High School	89 (78.1)	7 (46.7)	51 (44.3)	
Bachelor’s or equivalent	6 (5.3)	3 (20.0)	25 (21.7)	
Postgraduate	0 (0.0)	0 (0.0)	2 (1.7)	
Income, n (%)				0.035
<IDR 500.000	7 (6.1)	5 (33.3)	13 (11.3)	
IDR 500.000 - 2.500.000	71 (62.3)	4 (26.7)	63 (54.8)	
IDR 2.500.000 - 5.000.000	33 (28.9)	5 (33.3)	31 (27.0)	
IDR 5.000.000 - 10.000.000	3 (2.6)	1 (6.7)	6 (5.2)	
>IDR 10.000.000	0 (0.0)	0 (0.0)	2 (1.7)	
Hemoglobin level, gr%, median (IQR)	14.30[12.90, 15.67]	11.60[10.55, 13.65]	13.50[12.40, 14.90]	<0.001
CD4+ T cell counts,/μL, median (IQR)	853.00 [707.00, 1060.00]	289.00 [198.50, 353.50]	508.00 [353.00, 681.00]	<0.001
Skin Disease=Yes, n (%)	10 (8.8)	10 (66.7)	45 (39.1)	<0.001
Total Skincare Habit Score, median (IQR)	12.00 [10.00, 13.75]	11.00[8.00, 15.00]	12.00[10.00, 16.00]	0.361
HIV disease duration, median (IQR)	–	1.00 [1.00, 1.00]	8.00 [6.00, 12.00]	<0.001
Antiretroviral therapy (ART) treatment duration, median (IQR)	–	1.00 [1.00, 1.00]	8.00 [5.00, 12.00]	0.004

### 3.2 Alpha and beta diversity analysis

**Figure 1:**
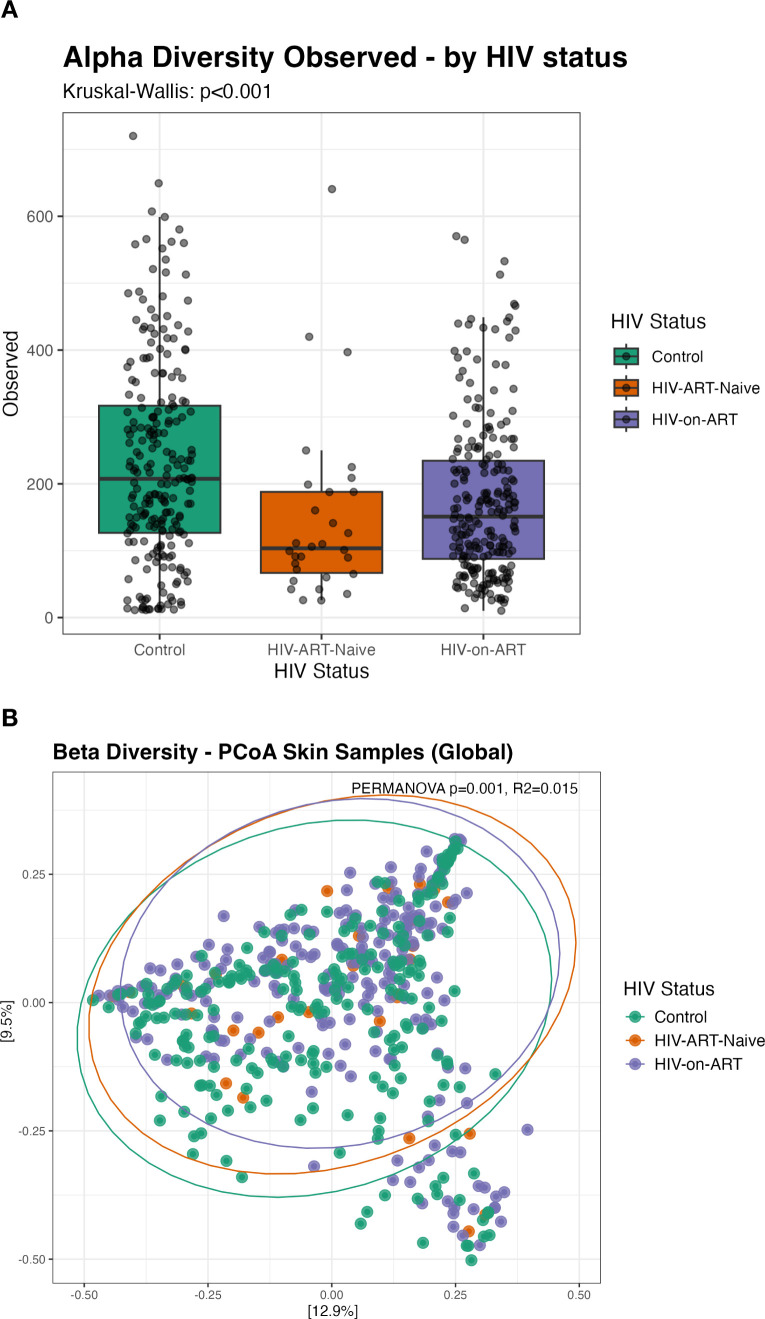
Comparison of skin microbial diversity and community composition among study cohorts. **(A)** Alpha diversity metrics: Observed diversity index, across healthy controls, HIV-ART-naïve, and HIV-on-ART. Boxplots represent the range and distribution of observed ASVs per sample. **(B)** PCoA based on Bray–Curtis dissimilarity visualizes sample-to-sample distances. Group differences in community composition were formally tested using PERMANOVA. Individual points correspond to samples; shaded ellipses show 95% confidence intervals. Separate PCoA plots for each cohort, highlighting distinct clustering of the skin microbiome in control (green), HIV-ART-naïve (orange), and HIV-on-ART (purple) groups.

Alpha diversity, assessed using the Observed index, was used to evaluate microbial richness across the three study groups (**Figure 1A**). Richness was significantly higher in the control group compared with both the HIV-ART-naïve and HIV-on-ART groups (Kruskal-Wallis p<0.001), indicating a modest reduction in microbial richness associated with HIV infection.

To assess overall differences in microbial community composition, beta diversity was examined using Principal Coordinate Analysis (PCoA) based on Bray–Curtis dissimilarity (**Figure 1B**). Visual inspection of the PCoA plots showed substantial overlap among groups, suggesting broadly comparable community structures. However, PERMANOVA identified a statistically significant difference in overall microbial composition across the three groups (p=0.001), although the effect size was small (R²=0.015), indicating limited separation at the community level.

**Figure 2:**
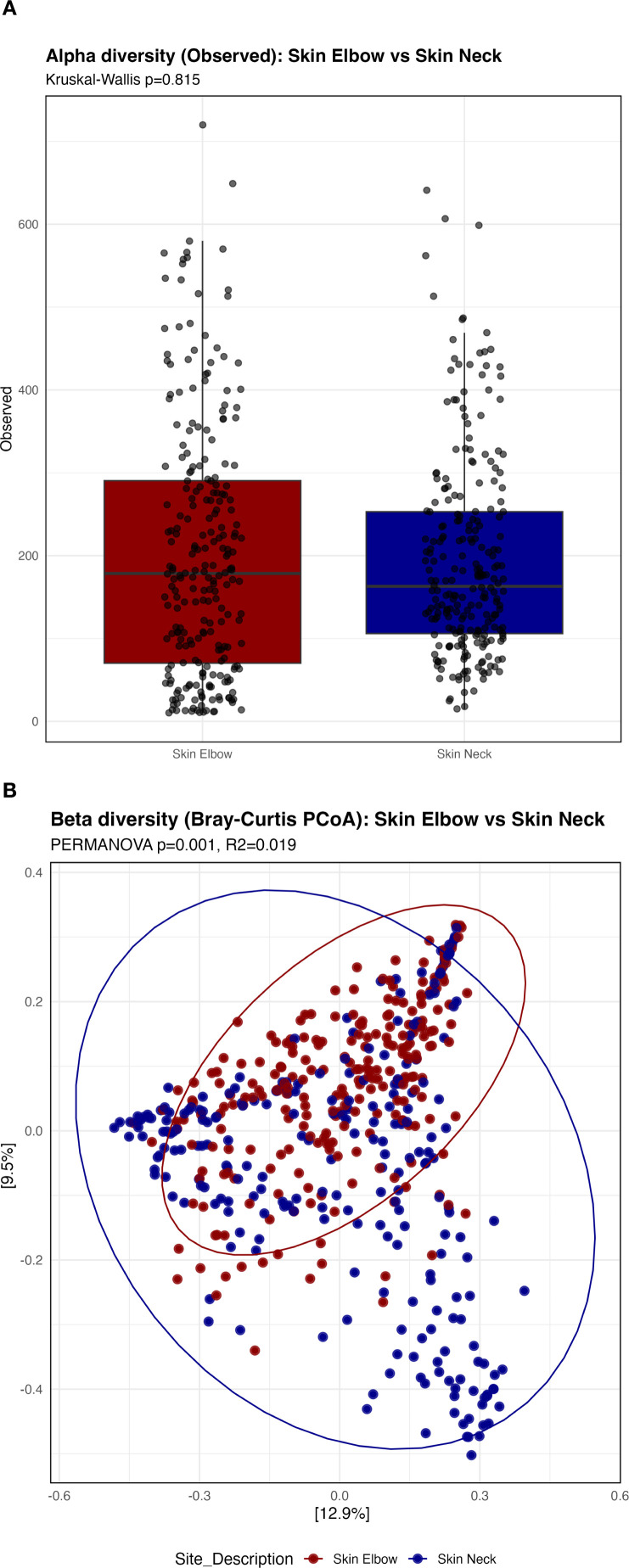
Alpha and beta diversity of the skin microbiome across different sampling sites. **(A)** Alpha diversity, assessed using observed ASV richness, is shown for samples collected from distinct skin sites. **(B)** Beta diversity, based on Bray–Curtis dissimilarity and visualized using principal coordinates analysis (PCoA), illustrates differences in overall microbial community composition between sites. Blue indicates samples obtained from the neck area, and red indicates samples obtained from the elbow area.

Given that samples were obtained from two distinct skin sites, we further examined site-specific differences in microbiome diversity and composition (**Figures 2A, B**). Alpha diversity did not differ significantly between elbow and neck samples, as measured by observed richness (p=0.815). In contrast, beta diversity analysis revealed significant site-related differences in microbial community composition. PCoA demonstrated greater dispersion of microbiome profiles in neck samples compared with elbow samples, with PERMANOVA confirming a significant site effect (R²=0.019, p=0.001), suggesting higher inter-individual variability at the neck site.

### 3.3 Relative abundance and differential abundance analysis

**Figure 3:**
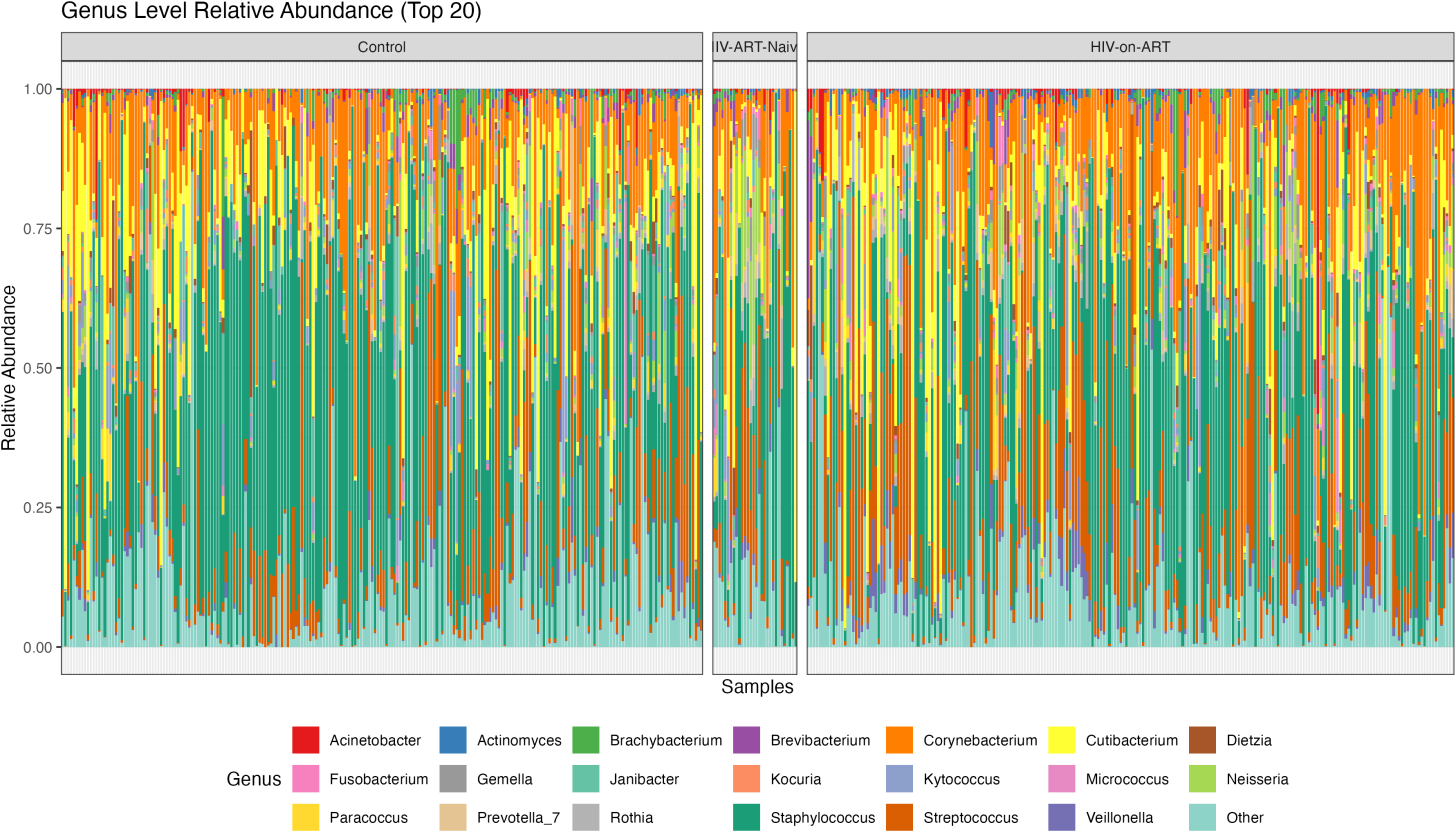
Relative abundance of the skin microbiome across study groups at the genus level. Stacked bar plots display the relative abundance of the top 20 bacterial genera for individual skin samples from controls, HIV-ART-naïve participants, and HIV-on-ART participants. The figure illustrates the distribution of dominant genera and the marked inter-individual variability in skin microbial community composition across study groups. Each bar represents one individual sample, and each color represents a bacterial genus.

**Figure 4:**
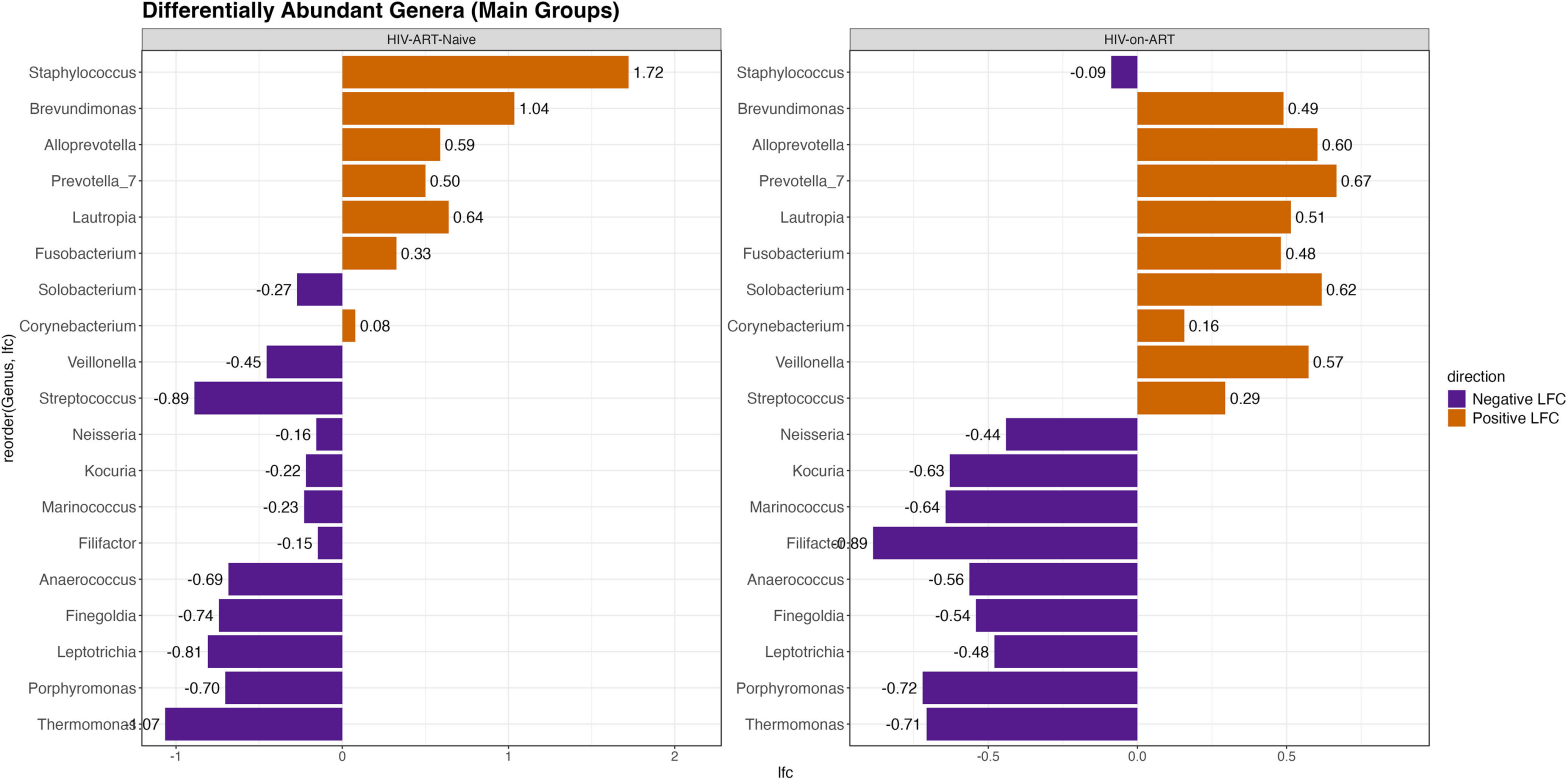
Differentially abundant skin microbial composition in HIV-ART-naïve and HIV-on-ART groups compared to control group. Waterfall plots illustrate the log fold changes (LFCs) of 19 genera that were found to differ significantly in abundance in both the HIV-ART-naïve and HIV-on-ART groups when compared with control group (ANCOM-BC, q<0.05). Bars represent average LFC values for each genus within each group. Color gradients indicate relative enrichment (yellow) or depletion (purple) compared with the control group.

The relative abundance plot (**Figure 3**) demonstrates broadly similar genus-level community structures across all study groups, with *Corynebacterium, Cutibacterium, Staphylococcus*, and *Streptococcus* consistently dominating the skin microbiome. Any between-group differences in relative abundance appear modest and should be interpreted cautiously given the compositional nature of these data. We therefore used genus-level differential abundance testing (ANCOM-BC2) to identify taxa associated with HIV status and ART exposure (**Figure 4**).

In the HIV-ART-naïve group, several genera showed marked reductions relative to controls, with the largest decrease observed for *Thermomonas* (LFC=−1.07), followed by *Streptococcus* (LFC=−0.89), *Leptotrichia* (LFC=−0.81), *Finegoldia* (LFC=−0.74), *Porphyromonas* (LFC=−0.70), and *Anaerococcus* (LFC=−0.69). In contrast, multiple genera were enriched in the HIV-ART-naïve group, most prominently *Staphylococcus* (LFC =+ 1.72), *Brevundimonas* (LFC =+ 1.04), *Lautropia* (LFC =+ 0.64), and *Alloprevotella* (LFC =+ 0.59), alongside smaller shifts in additional taxa.

In the HIV-on-ART group, increased abundance was primarily observed for *Prevotella_7* (LFC =+ 0.67), followed by *Alloprevotella*, *Solobacterium*, and *Veillonella*. The most pronounced reduction in this group was noted for *Filifactor* (LFC=−0.89), with additional decreases in *Porphyromonas*, *Thermomonas*, *Marinococcus*, and *Kocuria*. The remaining genera demonstrated relatively modest changes in either direction.

Notably, several genera exhibited opposing directions of differential abundance between the two HIV groups. *Staphylococcus* showed a positive log fold change in the HIV-ART-naïve group but a slight negative shift in the HIV-on-ART group (LFC=−0.09). Conversely, *Solobacterium*, *Veillonella*, and *Streptococcus* were reduced in the HIV-ART-naïve group yet increased in the HIV-on-ART group.

### 3.4 Determinants of skin microbiome variation

PERMANOVA analyses were performed to assess the extent to which clinical and baseline characteristics were associated with overall microbiome composition. In univariable models, all assessed variables showed statistically significant associations with community structure, although effect sizes were small (R² ranging from 0.005 to 0.019).

A multivariable PERMANOVA was then conducted to evaluate these factors jointly (**Table 2**). After adjusting for multiple covariates, HIV status remained a significant contributor to variation in skin microbiome composition (R²=0.009, p=0.01). Age (p=0.003), sex (p=0.001), BMI (p=0.002), sample type (p=0.001) and the total skin care habit score (p=0.004) were also statistically significant, though each explained a relatively small proportion of the overall variation. To aid interpretation of these multivariable findings, we visualized overall community structure using a Bray-Curtis PCoA plot with key metadata overlaid (**Supplementary Figure 1**).

**Table 2:** PERMANOVA analysis.

Variables	Univariable			Multivariable (n=452)		
	R^2^	F	P	R^2^	F	P
Age	0.006	2.896	0.001	0.004	2.062	0.003
Body Mass Index	0.008	4.065	0.001	0.005	2.582	0.002
Sex	0.014	7.030	0.001	0.015	7.131	0.001
Marriage	0.011	1.827	0.002			
Education	0.016	1.985	0.001			
Income	0.013	1.605	0.001			
Skin Diseases	0.005	2.453	0.002	0.003	1.298	0.129
Total Skin Care Habit score	0.005	2.272	0.001	0.005	2.195	0.004
Sample type (neck vs elbow)	0.019	9.215	0.001	0.017	8.255	0.001
CD4+ T cells counts	0.005	2.479	0.001	0.002	0.892	0.623
HIV status	0.015	3.681	0.001	0.009	2.227	0.001

In the relative abundance analyses (**Supplementary Figure 2**) stratified by BMI categories, beyond the overall dominance of *Cutibacterium, Staphylococcus*, and *Streptococcus*, the low-BMI group exhibited a higher relative abundance of *Streptococcus* compared with the high-BMI group. Across skincare habit score categories, a similar pattern was observed, with higher *Streptococcus* abundance detected in the high skincare score group. With respect to sample type, elbow skin samples showed greater dominance of *Corynebacterium, Staphylococcus*, and *Streptococcus*, whereas neck skin samples were characterized by higher relative abundance of *Cutibacterium* and *Staphylococcus.*

We further performed subgroup analyses to explore microbiome differences according to key variables identified in the multivariable model, namely BMI, skincare habit score, and sample type (**Supplementary Figure 3**).

When stratified by BMI, HIV-associated differences in skin microbiome composition demonstrated both shared patterns across BMI categories and BMI-dependent variations. In individuals with higher BMI, there was a relative enrichment of classical skin commensals, most notably *Staphylococcus, Corynebacterium*, and *Cutibacterium*, compared with the lower-BMI group. In contrast, lower BMI was associated with enrichment of *Bulleidia, Pseudoramibacter, Eikenella*, and *Capnocytophaga*. When further divided into subsets focusing on HIV-on-ART group, enrichment of *Staphylococcus, Brevibacterium*, and *Mycobacterium* was observed, alongside depletion of *Eikenella, Cryptobacterium*, and *Cantonella*.

Stratification based on skincare habit score was performed by dividing participants into high and low score groups using a median cutoff of 12. In the high skincare habit score group, moderate enrichment of skin-associated genera was observed, most notably *Cutibacterium, Streptococcus*, and *Acinetobacter* (LFC + 0.50 to +0.59). In contrast, a pronounced depletion of *Bulleidia, Kingella*, and *Eikenella* was noted (LFC −1.10, −0.94, and −0.85, respectively). Within the HIV-on-ART subset, enrichment of *Streptococcus, Thermomonas*, and *Escherichia–Shigella* was observed, accompanied by depletion of *Aggregatibacter* and *Eikenella.*

Finally, when stratified by sample type, elbow skin samples showed a more pronounced increase in *Streptococcus, Neisseria*, and *Gemella* compared with neck skin samples (LFC + 0.77, +0.73, and +0.60, respectively). In parallel, neck skin samples exhibited greater enrichment of *Bulleidia* and *Pseudoramibacter.* Within the HIV-on-ART subset, enrichment of *Streptococcus, Marinilutecoccus*, and *Jeotgalicoccus* was observed, while depletion of *Tsukamurella* and *Eikenella* was noted.

## 4 Discussion

Baseline characteristics differed significantly across groups and are important for interpreting the skin microbiome findings. HIV-ART-naïve individuals were younger, had lower BMI, and showed marked immunologic impairment compared with those on ART and healthy controls, consistent with untreated HIV infection. Socioeconomic differences across groups may also reflect variation in lifestyle and hygiene-related factors that can influence skin microbial composition (Hou et al., 2022; Nguyen et al., 2025).

Immunologic and hematologic markers improved stepwise from HIV-ART-naïve individuals to those on ART and controls, indicating immune restoration with therapy, although residual differences persisted. Skin disease prevalence followed a similar gradient, being highest in HIV-ART-naïve participants and lowest in controls. These patterns underscore the close relationship between immune status, skin health, and the skin microbiome, and justify adjustment for demographic and clinical covariates in subsequent analyses (Liu et al., 2023).

Alpha diversity was reduced in both HIV groups compared with healthy controls, indicating lower skin microbial richness associated with HIV infection that was not fully normalized by ART. This suggests persistent alterations in skin microbial composition, possibly related to residual immune dysfunction despite treatment (Oh et al., 2013). Beta diversity analyses revealed statistically significant, but small, differences in overall community composition between groups. The absence of clear separation in PCoA plots and the low PERMANOVA R² indicate that HIV status contributes only modestly to overall variation of skin microbiome, with interindividual and environmental factors likely playing a larger role (Ogai et al., 2023; Anshory et al., 2025).

Sampling site influenced community structure, with greater variability in community composition observed in neck samples compared with the elbow, despite similar richness. This likely reflects anatomical and microenvironmental differences and underscores the importance of accounting for sampling site in skin microbiome analyses (Ogai et al., 2018; Santiago-Rodriguez et al., 2023). Notably, consistent with established site-specific skin microenvironmental differences, sampling site explained a larger proportion of variance in community composition than HIV status (Ogai et al., 2023). However, HIV status remained independently associated with microbiome composition after adjustment, indicating disease-related effects that occur within the constraints of site-specific microbial communities.

### 4.1 Patterns in HIV-associated skin microbiome changes

At the genus level, the overall skin microbiome composition was broadly conserved across study groups, with *Corynebacterium, Cutibacterium, Staphylococcus*, and *Streptococcus* consistently dominating. This relative compositional stability suggests that HIV infection does not result in a complete restructuring of the skin microbiome, but rather in more subtle, genus-specific alterations (Ogai et al., 2023; Anshory et al., 2025). This supports the interpretation that HIV-associated differences represent targeted shifts within stable site-dependent communities rather than global dysbiosis.

Differential abundance analyses revealed genus-specific alterations that were not readily apparent from relative abundance profiles alone, highlighting more subtle but biologically relevant shifts in the skin microbiome associated with HIV status and ART exposure. In ART-naïve individuals, the pronounced enrichment of *Staphylococcus*, together with concurrent reductions in several anaerobic and oral-associated genera such as *Porphyromonas, Anaerococcus, Finegoldia*, and *Leptotrichia*, suggests a redistribution among key skin-associated taxa rather than a uniform loss of microbial diversity. This pattern may reflect selective pressures related to immune dysfunction, altered skin barrier integrity, or changes in the cutaneous microenvironment, as reported in other inflammatory and immune-mediated skin diseases (Eribe and Olsen, 2017; Piazzesi et al., 2024). The concurrent depletion of anaerobic or mucosal-associated genera and enrichment of classical skin commensals suggests a narrowing of ecological niches on the skin under immune compromise, potentially favoring stress-resilient taxa and contributing to altered host–microbe interactions in untreated HIV, as also observed in other immunodeficient subjects (Oh et al., 2013). Although effect sizes were modest, such consistent shifts may still be clinically relevant, given the roles of dominant skin commensals in maintaining the skin barrier, limit pathogen overgrowth, and shape local immune and inflammatory responses (Lee and Kim, 2022). These findings suggest that HIV-associated immune dysfunction may alter local cutaneous niches, increasing susceptibility to dermatologic infections and inflammatory dermatoses in people living with HIV, even in the absence of overt dysbiosis.

In individuals receiving ART, differential abundance patterns were generally attenuated, with smaller effect sizes and a redistribution of taxa distinct from the HIV-ART-naïve group. The near-neutralization of *Staphylococcus* abundance and the enrichment of genera such as *Prevotella_7*, *Solobacterium*, and *Veillonella* suggest partial restoration and remodeling of the skin microbiome following viral suppression, consistent with microbiome ‘normalization’ reported after effective therapy in other disease contexts (Kehrmann et al., 2023; Che et al., 2025). However, the persistence of reduced abundance in genera such as *Filifactor, Porphyromonas*, and *Thermomonas* indicates that ART does not fully align the microbial composition with that observed in HIV-negative individuals. Together, these findings support partial attenuation rather than complete normalization with ART.

Importantly, several genera displayed opposing directions of differential abundance between HIV-ART-naïve and HIV-on-ART individuals, highlighting heterogeneity in microbiome composition across treatment status. These patterns indicate that ART-treated individuals exhibit microbial compositions that differ from those of untreated individuals, potentially reflecting differences in immune status and host or environmental factors. Collectively, these findings suggest that ART is associated with partial attenuation of HIV-related microbiome alterations, while genus-specific differences remain evident on the skin. Furthermore, HIV-associated genus-level differences were observed within both sebaceous and dry sites, indicating that the HIV-related signal is not restricted to a single skin microenvironment but emerges within a strongly site-structured microbial landscape.

### 4.2 Multivariable determinants of skin microbiome composition in HIV infection

Multivariable PERMANOVA analyses indicate that skin microbiome composition is influenced by a combination of HIV status and host or environmental factors, each contributing modestly to overall community variation (Ogai et al., 2023). Although effect sizes were small, HIV status remained independently associated with microbiome composition after adjustment, supporting a direct but subtle influence of HIV infection on the skin microbial ecosystem. Age, sex, BMI, sampling site, and skincare habits were also significant contributors, underscoring the multifactorial regulation of the skin microbiome (Anshory et al., 2025).

Subgroup analyses provided additional context for these associations. Across BMI strata, HIV infection was consistently linked to enrichment of classical skin commensals, particularly *Corynebacterium* and *Staphylococcus*, alongside depletion of *Streptococcus* and other anaerobic or mucosa-associated genera, in line with previous reports. Differences between BMI categories suggest that host metabolic status may modulate the magnitude and specific pattern of HIV-associated microbial shifts (Brandwein et al., 2019).

Skincare habits appeared to modify HIV-associated skin microbiome differences. Higher skincare engagement was associated with a microbial profile more enriched in skin-adapted taxa and reduced representation of oral- or mucosa-associated genera, suggesting that skincare practices may partially buffer HIV-related microbial imbalance. However, these effects did not fully normalize community composition, supporting the notion that hygiene practices influence the skin microbiome without overriding underlying host or disease-related factors (Kortekaas Krohn et al., 2024).

Anatomical site also influenced skin microbiome composition, with distinct microbial patterns observed between elbow and neck skin. These site-dependent differences are consistent with established evidence that local skin microenvironments shape microbial communities and persist even in the context of systemic immune alterations such as HIV infection and ART (Grice et al., 2009; Cho et al., 2025). This highlights a potential interaction framework: HIV and ART effects may operate within each skin microenvironment, modulating taxa abundance without displacing the core ecological structure determined by local microenvironmental conditions.

### 4.3 Study strengths and limitations

A key strength of this study is the well-characterized cohort with parallel sampling from two anatomical sites and comprehensive adjustment for demographic, clinical, and behavioral factors, allowing robust assessment of independent determinants of skin microbiome composition.

However, several limitations should be acknowledged. The cross-sectional design precludes causal inference and limits evaluation of temporal changes in the skin microbiome associated with HIV infection or ART initiation. In addition, the relatively small number of HIV-ART-naïve participants may have reduced statistical power for this subgroup, particularly for low-abundance taxa.

Although multivariable models adjusted for major demographic and clinical factors, residual confounding from unmeasured variables such as dietary factors, environmental exposures, detailed hygiene practices, topical treatments, and prior antimicrobial use remains possible because these factors were not collected in this study. Effect sizes for HIV status and other covariates were modest, reflecting high inter-individual variability in the skin microbiome, and should be interpreted as subtle ecological shifts rather than overt dysbiosis. Finally, the use of 16S rRNA gene sequencing limits taxonomic and functional resolution.

Future studies incorporating longitudinal designs and integrative functional approaches, including metagenomics, proteomics, and metabolomics, are warranted to elucidate the temporal dynamics, functional consequences, and clinical relevance of HIV- and ART-associated skin microbiome alterations. Specifically, linking taxa-level shifts to host markers of epidermal barrier integrity and cutaneous inflammation may clarify their mechanistic and clinical significance.

## 5 Conclusions

This study shows that HIV status is associated with subtle, but consistent, differences in skin microbiome diversity and genus-level composition, with partial attenuation observed in individuals receiving ART. These findings indicate targeted ecological shifts rather than global disruption of the skin microbial community. Overall, our findings suggest that ART is associated with a distinct, but not fully normalized, skin microbiome profile, highlighting the need for longitudinal and functional studies to better understand the clinical relevance of persistent microbiome alterations in people living with HIV. Importantly, these HIV-associated shifts occurred within the context of strong site-specific skin microenvironments, with sampling site explaining more variance than HIV status. Future longitudinal studies should integrate functional profiling and correlate specific microbial shifts with biomarkers of epidermal barrier integrity and cutaneous inflammation, and ideally with subsequent dermatologic outcomes.

## Data Availability

The datasets presented in this article are not readily available because of legal restrictions under the Material/Data Transfer Agreement of the Ministry of Health of the Republic of Indonesia. Requests to access the datasets should be directed to muhammadanshory@ub.ac.id.
