# Supplementary material for: Skin microbiome variation in people living with HIV: associations with antiretroviral therapy and host factors
Source: Front Cell Infect Microbiol. 2026 Apr 15;16:1794878. doi: 10.3389/fcimb.2026.1794878 (PMC13124997; doi:10.3389/fcimb.2026.1794878)
Supplement: Supplementary file 1 [file Table1.docx]

Supplementary Material

# Supplementary Figures and Tables

## Supplementary Tables

**Supplementary Table 1. Skincare habit score**

| **Questionnaire items** | **Responses** | **Score** |
| --- | --- | --- |
| **Use of skin lotion or skincare products** | No | **0** |
|  | Yes | **1** |
| **Frequency of face washing** | Don’t know | **0** |
|  | Once per week | **1** |
|  | 2–3 times per week | **2** |
|  | Once per day | **3** |
|  | 2–3 times per day | **4** |
| **Frequency of bathing** | Don’t know | **0** |
|  | Once per week | **1** |
|  | 2–3 times per week | **2** |
|  | Once per day | **3** |
|  | 2–3 times per day | **4** |
| **Frequency of shampooing** | Don’t know | **0** |
|  | Once per week | **1** |
|  | 2–3 times per week | **2** |
|  | Once per day | **3** |
|  | 2–3 times per day | **4** |
| **Frequency of using leave-on products (e.g., moisturizer) on face and neck** | Never | **0** |
|  | Once per week | **1** |
|  | 2–3 times per week | **2** |
|  | Once per day | **3** |
|  | Don’t know | **0** |
| **Frequency of using leave-on products (e.g., moisturizer) on body** | Never | **0** |
|  | Once per week | **1** |
|  | 2–3 times per week | **2** |
|  | Once per day | **3** |
|  | Don’t know | **0** |
| **Total maximum score** | | **19** |

## Supplementary Figures

#
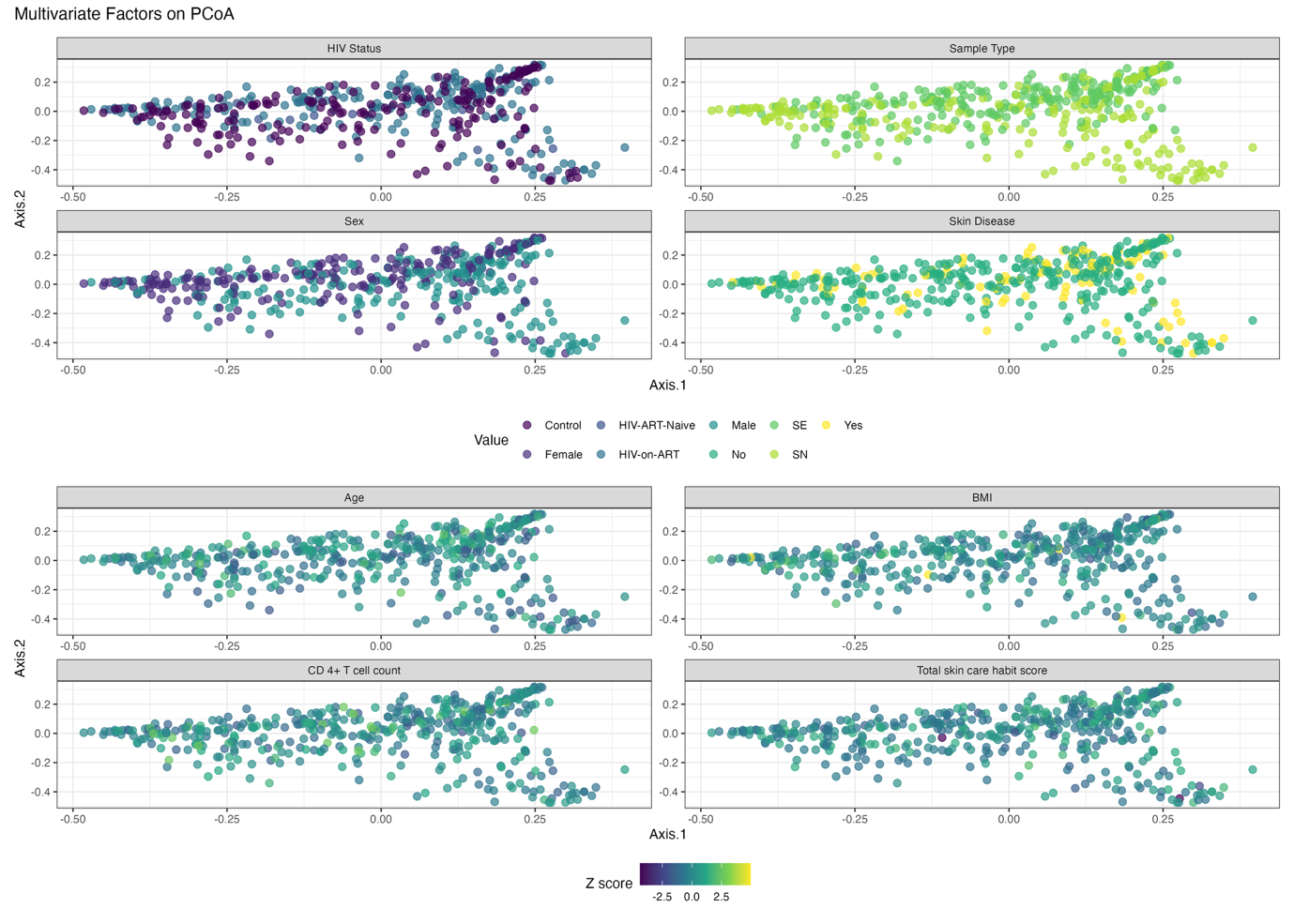


# Supplementary Figure S1. Multivariate factors on PCoA. PCoA of Bray-Curtis dissimilarities for skin microbiome samples (Axes 1–2). Points represent samples. Top panel: categorical variables colored by HIV Status, Sample Type, and Skin Disease. Bottom panel: continuous variables colored by z scores for CD4+ T cell counts, Age, Total skin care habit score, and BMI (standardized within each variable).

# A.

#
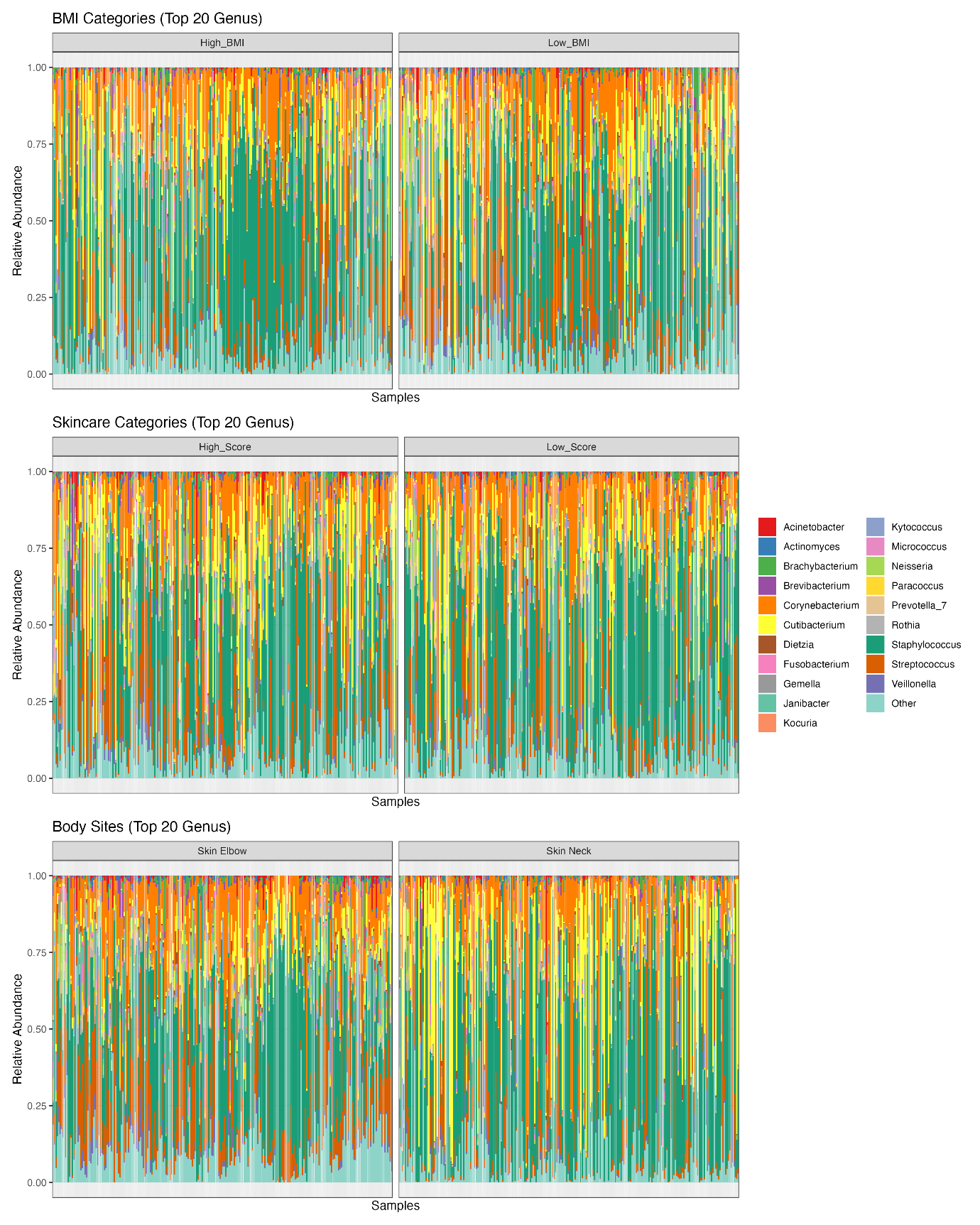


# B.

#
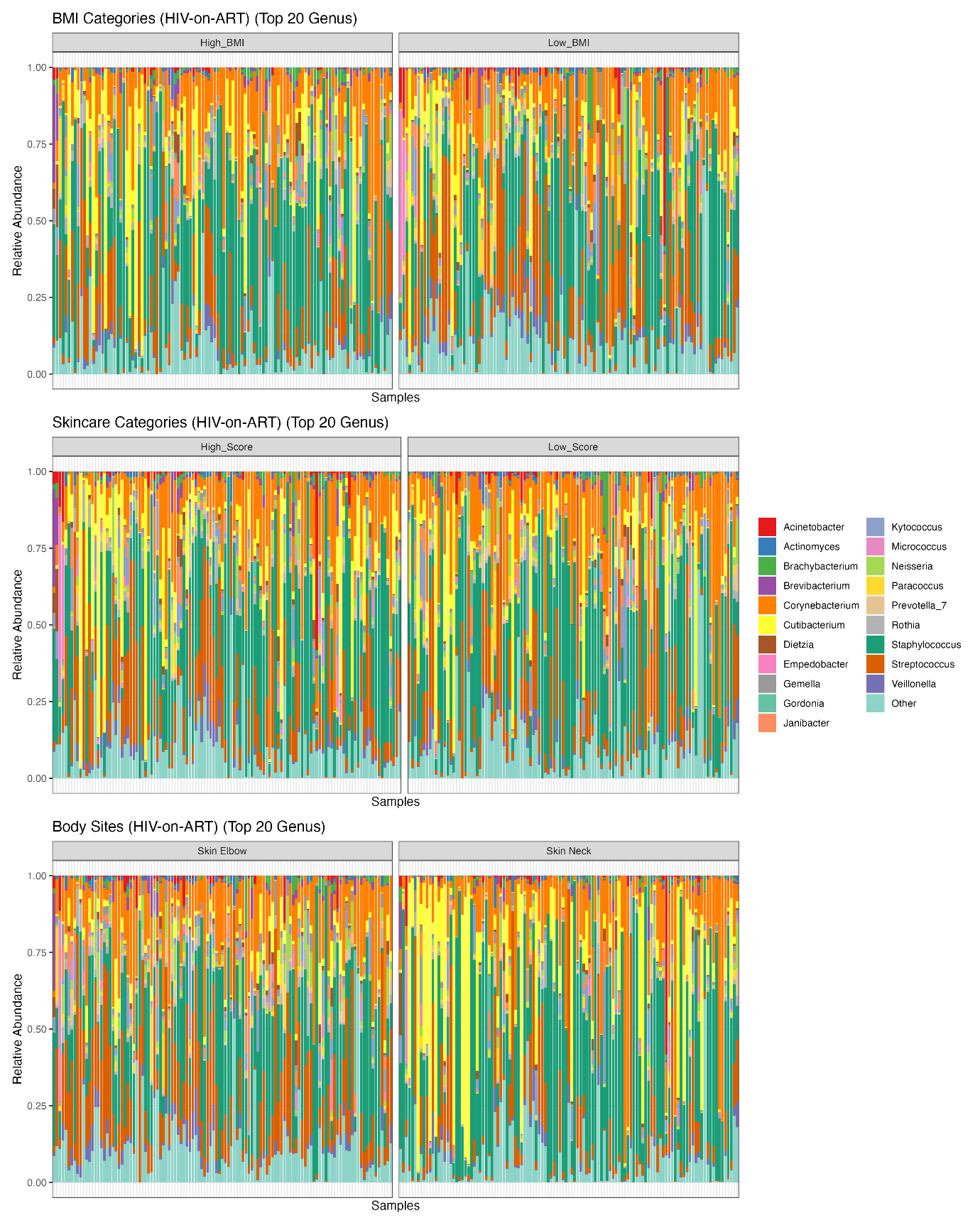


# Supplementary Figure S2. Relative abundance of the skin microbiome at the genus level stratified by key variables. Stacked bar plots depict genus-level relative abundances stratified by BMI, skincare habit score, and sample type for (A) the full study population and (B) participants living with HIV receiving ART. Each bar represents an individual participant, with colors denoting different bacterial genera. The figure illustrates the distribution of dominant genera and inter-individual variability in skin microbial community composition across strata.

# A.

#
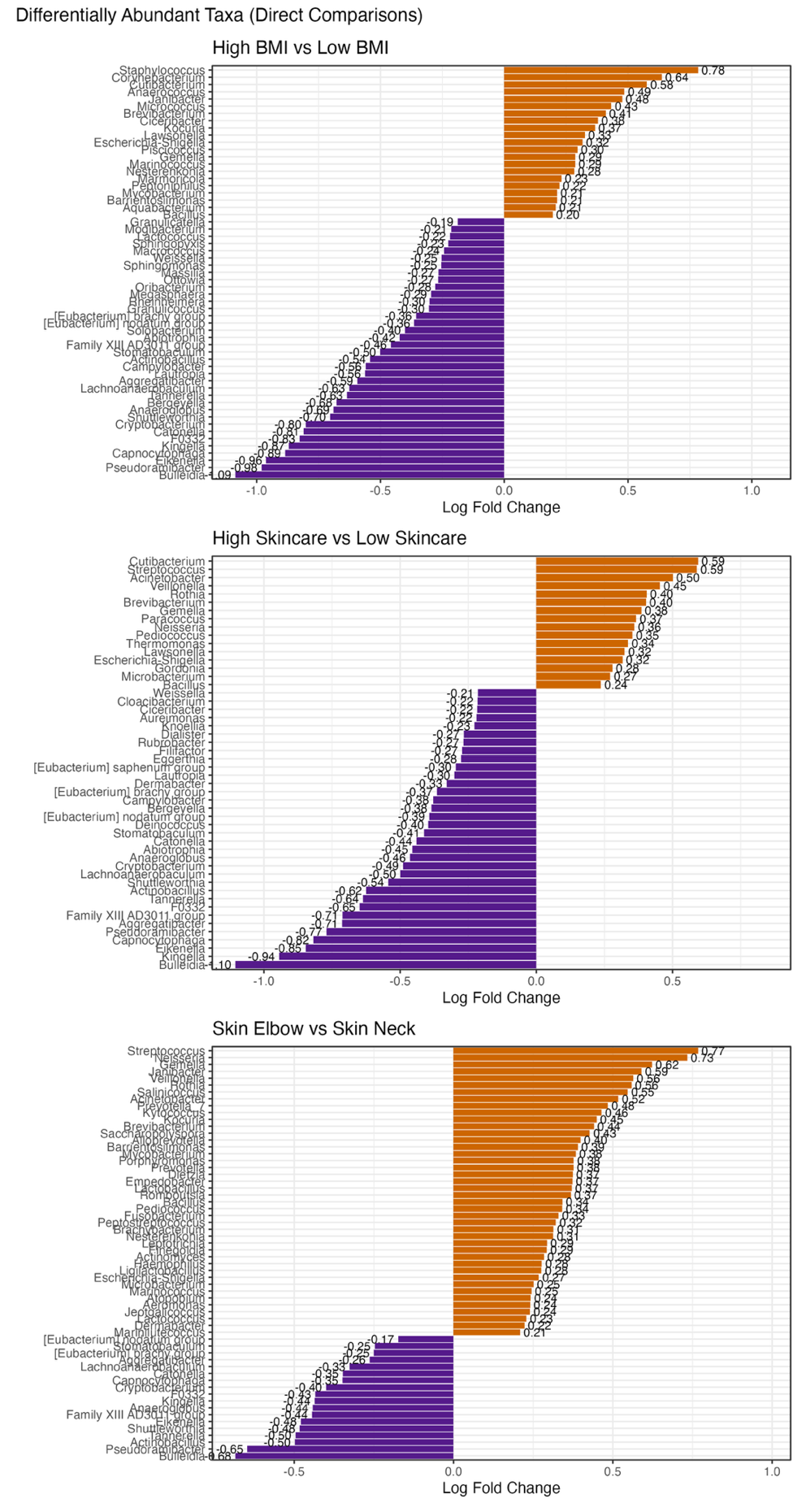


# B.

#
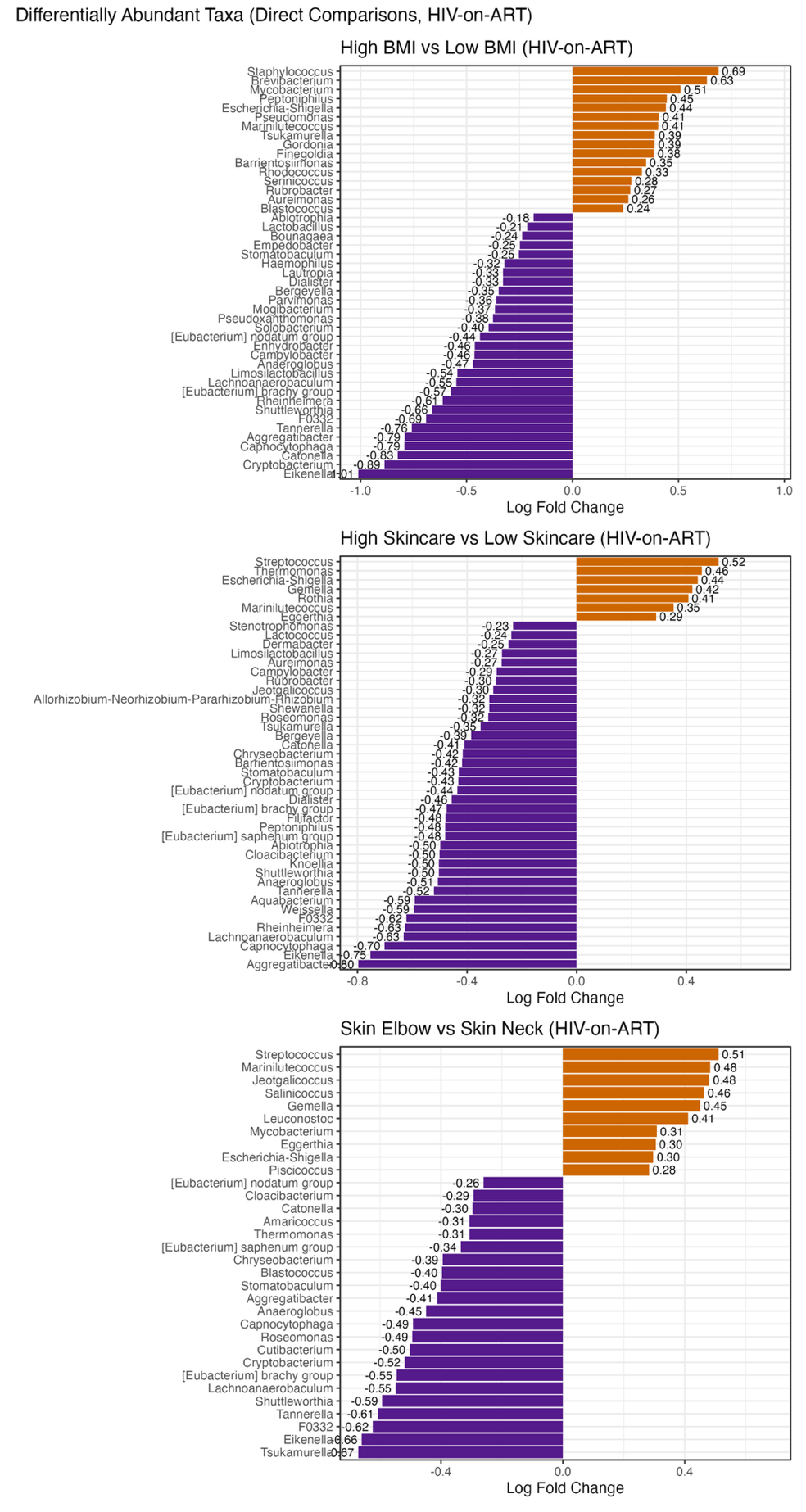


# Supplementary Figure S3. Differentially abundant skin microbiome genera identified across multivariable models. Waterfall plots show estimated log fold changes (LFCs) for bacterial genera identified as differentially abundant using ANCOM-BC in models incorporating BMI, skincare habit score, and sample type for (A) the full study population and (B) participants living with HIV receiving ART. All models were adjusted for BMI, skincare habit score, and sample type. Color gradients indicate relative enrichment (dark yellow) or depletion (dark purple).
